# Impact of transcutaneous neuromuscular electrical stimulation or resistance exercise on skeletal muscle mRNA expression in COPD

**DOI:** 10.2147/COPD.S189896

**Published:** 2019-06-28

**Authors:** Lorna E Latimer, Despina Constantin, Neil J Greening, Lori Calvert, Manoj K Menon, Michael C Steiner, Paul L Greenhaff

**Affiliations:** 1 Department of Respiratory Sciences, University of Leicester, Leicester, UK; 2 Institute for Lung Health, National Institute for Health Research (NIHR) Leicester Biomedical Research Centre - Respiratory, Glenfield Hospital, Leicester, UK; 3 Medical Research Council/Arthritis Research UK (MRC/ARUK) Centre for Musculoskeletal Ageing Research, University of Nottingham, Nottingham, UK; 4 Centre for Sport, Exercise and Osteoarthritis Research, University of Nottingham, Nottingham, UK; 5 NIHR Nottingham Biomedical Research Centre, University of Nottingham, Nottingham, UK; 6 Peterborough and Stamford Hospitals NHS Foundation Trust, Peterborough City Hospital, Bretton, UK; 7 Barking, Havering and Redbridge University Hospitals NHS Trust, Chest Clinic, King George Hospital, Ilford, UK

**Keywords:** COPD, NMES, skeletal muscle, gene expression, resistance exercise

## Abstract

**Background:** Voluntary resistance exercise (RE) training increases muscle mass and strength in patients with chronic obstructive pulmonary disease (COPD). Nonvolitional transcutaneous neuromuscular electrical stimulation (NMES) may be an alternative strategy for reducing ambulatory muscle weakness in patients unable to perform RE training, but little comparative data are available. This study, therefore, investigated changes in muscle mRNA abundance of a number of gene targets in response to a single bout of NMES compared with RE.

**Methods:** Twenty-six patients with stable COPD (15 male; FEV_1_, 43±18% predicted; age, 64±8 years; fat free mass index, 16.6±1.8 kg/m^2^) undertook 30 minutes of quadriceps NMES (50 Hz, current at the limit of tolerance) or 5×30 maximal voluntary isokinetic knee extensions. Vastus lateralis muscle biopsies were obtained at rest immediately before and 24 hours after intervention. Expression of 384 targeted mRNA transcripts was assessed by real time TaqMan PCR. Significant change in expression from baseline was determined using the ΔΔC_T_ method with a false discovery rate (FDR) of <5%.

**Results:** NMES and RE altered mRNA abundance of 18 and 68 genes, respectively (FDR <5%), of which 14 genes were common to both interventions and of the same magnitude of fold change. Biological functions of upregulated genes included inflammation, hypertrophy, muscle protein turnover, and muscle growth, whilst downregulated genes included mitochondrial and cell signaling functions.

**Conclusions:** Compared with NMES, RE had a broader impact on mRNA abundance and, therefore, appears to be the superior intervention for maximizing transcriptional responses in the quadriceps of patients with COPD. However, if voluntary RE is not feasible in a clinical setting, NMES by modifying expression of genes known to impact upon muscle mass and strength may have a positive influence on muscle function.

## Introduction

Impaired skeletal muscle function is a common systemic feature of COPD and an important contributor to morbidity and mortality.^1^,^2^ There is substantive evidence demonstrating the benefits of resistance exercise (RE) training in COPD,^3^–^5^ and it is currently recommended that this modality of training should be incorporated into pulmonary rehabilitation programs.^6^ However, not all patients with COPD are able to perform conventional voluntary resistance training of sufficient intensity to bring about meaningful gains in muscle mass and strength, due to advanced deconditioning, acute exacerbation, and associated dyspnoea or hospitalization. Transcutaneous neuromuscular electrical stimulation (NMES) is a nonvolitional means of evoking muscle contraction that places minimal demand on the cardiorespiratory system, and does not induce significant dyspnoea.^7^,^8^ For these reasons, NMES may be an effective strategy for the maintenance or improvement of muscle function in settings as above, where voluntary RE training cannot be performed.^9^

Despite eliciting low contractile forces during training (typically below 15% of maximal voluntary isometric strength^10^), clinical studies have suggested that NMES increases muscle mass and strength in COPD.^11^,^12^ To date, however, understanding of the training adaptations to NMES at a muscle level, and how these compare to those elicited by voluntary RE is limited. It is known that an unaccustomed bout of voluntary RE in COPD causes a change in expression of a wide range of mRNA transcripts,^13^ but this information is lacking for NMES, where it is likely that a smaller muscle mass will be recruited.

This study investigated changes in the expression of muscle mRNA transcripts 24 hours following a single bout of NMES or RE in matched cohorts of patients with COPD. Specifically, we investigated whether the abundance of gene transcripts shown to be responsive to RE in healthy, young volunteers^14^,^15^ followed similar patterns of change when comparing NMES and RE, and whether the magnitude of any change was similar for the two contraction modalities.

## Methods

### Subjects

Patients with a clinical diagnosis of COPD, confirmed airflow obstruction (FEV_1_<80% predicted, FEV_1_/FVC ratio <0.7) and significant self-reported exertional dyspnoea (MRC Grade ≥3) were recruited from outpatient clinics, pulmonary rehabilitation waiting lists, and previous research volunteers at the University Hospitals of Leicester NHS Trust, UK. At the time of recruitment, patients were free from exacerbation and oral steroid medication for ≥4 weeks, had not attended pulmonary rehabilitation for ≥1 year, and had no comorbidities leading to significant exercise limitations.

### Experimental protocol

Matched cohorts of patients with COPD undertaking NMES and RE were recruited to the study. Patients attended baseline assessments to perform spirometry, undergo body composition measures, and perform NMES or RE familiarization a minimum of 1 week before the first biopsy visit. Resting biopsies were performed on the *vastus lateralis* muscle using the micro-biopsy technique previously used in our laboratory.^16^ Tissue was snap frozen in liquid nitrogen and stored for later analysis. After tissue acquisition, a light dressing was applied to the biopsy site, and a single exercise bout (either transcutaneous NMES or voluntary RE of the quadriceps) was performed. Twenty-four hours later, a second resting biopsy was performed at least 2.5 cm from the previous biopsy site, thereby minimizing confounding changes in mRNA abundance due to tissue sampling.^13^,^14^,^17^ Previous work has shown expression of genes related to skeletal muscle mass regulation is altered 24 hours post-RE in COPD and health.^13^,^14^

Subjects for this study were drawn from two cohorts who undertook a NMES or RE intervention in otherwise identical experimental designs. Groups were matched based on lung function and body composition (Table 1). This study was conducted in accordance with the Declaration of Helsinki; ethical approval was granted by the UK National Health Service (NHS) Research Ethics Committee (REC) (NMES Study: West Midlands REC, reference 10/H1208/73; RE Study: Leicestershire, Northamptonshire and Rutland REC, reference 05/Q2502/131), and participants provided written informed consent.

**Table 1:** Patients’ baseline characteristics

Variable	NMES	Voluntary RE	*P*
Males/females, n	7/6	8/5	0.691
Age, years	63.6 (9.1)	64.2 (7.0)	0.867
Height, m	1.61 (9)	1.65 (11)	0.351
Body mass, kg	66.4 (12.5)	65.4 (20.5)	0.883
BMI, kg/m^2^	25.6 (4.2)	23.7 (5.6)	0.341
FFMI, kg/m^2^	16.8 (1.7)	16.4 (2.0)	0.700
FEV_1_, l	1.02 (0.34)	1.02 (0.47)	0.996
FEV_1_, % predicted	45.5 (19.3)	40.3 (16.7)	0.476
FEV_1_/FVC	0.41 (0.10)	0.44 (0.13)	0.552
Smoking status (current/ex/never)	1/11/1	7/6/0	0.031
Pack years smoked	44.3 (23.5)	56.2 (35.8)	0.325
MRC grade	4 (3–4)	4 (3–4)	0.511

**Notes: **Values are mean (SD) except MRC Grade which is median (IQR). **Abbreviations: **BMI, body mass index; FFMI, fat free mass index (by DEXA scan); FEV_1_, forced expiratory volume in 1 s; FVC, forced vital capacity; MRC Grade, Medical Research Council dyspnoea scale; NMES, transcutaneous neuromuscular electrical stimulation; RE, resistance exercise.

### Baseline assessments

Height and body mass in light clothing were measured before body composition assessment using dual energy x-ray absorptiometry (DEXA; Lunar Prodigy, GE, Buckinghamshire, UK). Body Mass Index (BMI) was calculated as: total body mass (kg)/height (m)^2^. Fat Free Mass Index (FFMI) was calculated using the same equation, but with body mass replaced with whole-body lean mass+whole-body bone mineral mass (kg). Spirometry was performed using a portable spirometer in accordance with British Thoracic Society guidelines.^18^

### Exercise protocol

The NMES protocol employed a hand-held, battery powered device (Empi 300PV, MN, USA) connected to two skin surface gel electrodes placed over the quadriceps (see Supplementary materials for details). The protocol consisted of 30 minutes of stimulation with a biphasic pulse at 50 Hz (pulse duration 300 µs) made up of 15-second duty cycles with 5-second rests. Patients self-selected stimulation intensity and were encouraged to set the current at the limit of tolerability. During familiarization, patients underwent stimulation starting at minimum intensity (1 mA) and progressing to the self-determined limit of tolerance during a session lasting no longer than 10 minutes.

RE consisted of five sets of 30 maximal isokinetic knee extensions at 180°/s with 1 minute rest between sets performed on an isokinetic dynamometer (Cybex NORM II, CSMi, Stoughton, USA). Patients were seated with hip and knee flexion of 90°. This protocol has previously been shown to change quadriceps mRNA abundance in COPD patients 24 hours following exercise.^13^

### RNA extraction

RNA was extracted from muscle samples using TRI Reagent (Applied Biosystems/Life Technologies, Paisley, UK) and reverse transcribed using SuperScript III (Life Technologies/Invitrogen) to synthesize complimentary DNA (see Supplementary materials). Complimentary DNA was loaded onto TaqMan 384 well custom Low Density Array (LDA) microfluidic cards (Applied Biosystems/Life Technologies) and 40 cycles of automated polymerase chain reaction (PCR) performed on a TaqMan 7900HT Real-Time PCR Instrument (Applied Biosystems, Paisley, UK).

### Muscle mRNA expression

The abundance of mRNA was assessed using automated 384-well, LDA cards. The microfluidic cards were custom-designed to target families of genes whose functions include metabolic processes, mitochondria, diabetes, cell cycle/growth/differentiation, inflammation, and immune responses. Target selection was directed by data from two studies that employed a similar isokinetic RE protocol to that used in the current study. The first study reported an Affymetrix based analysis of tissue sampled at rest pre- and 24 hours-post resistance exercise in healthy volunteers.^14^ The second study highlighted gene expression changes following a similar bout of RE in young healthy volunteers who had undergone a period of immobilization.^15^

### Data analysis

Gene expression data were analyzed using the comparative C_T_ method (ΔΔC_T_), which permits relative quantification of the target gene transcript against an internal control gene transcript.^19^ A suitable control gene (*hydroxymethylbilane synthase*; *HMBS*) was selected that had stable C_T_ values across time points. Paired *t*-tests were used to identify significant change in expression of the target gene relative to *HMBS *(ΔC_T_) from baseline to 24 hours, and the False Discovery Rate (FDR) adjustment applied to control for multiple comparisons using the R Statistical Package (R Version 3.0.0, 2013–04-03, The R Foundation for Statistical Computing). Expression values are presented as fold change from baseline (2^−ΔΔCT^) and significant within-group change was defined by a FDR <5%. Missing values occurred where gene expression was below the limit of detection after 40 cycles of PCR. A gene was excluded from the analysis if there were more than two missing data points. Between-group differences in physiological variables were tested by *t*-test, Mann-Whitney U-test (ordinal data), or Pearson Χ^2^ test (categorical data).

## Results

### Patient characteristics

Thirteen COPD patients received NMES and 13 patients performed voluntary RE. There were no significant differences between the groups for baseline measures of lung function, MRC grade, or body composition, although there was a significant difference between the groups for smoking history, with the RE group containing more current smokers (Table 1). 

### Exercise bout

The mean (SD) peak torque generated during RE was 38 (±13) Nm, and the mean work done over five sets of 30 isometric knee extensions by the voluntary RE group was 2482 (±925) J. The mean (SD) electrical stimulation current during NMES was 39.3 (±12.7) mA.

### mRNA expression

**Figure 1:**
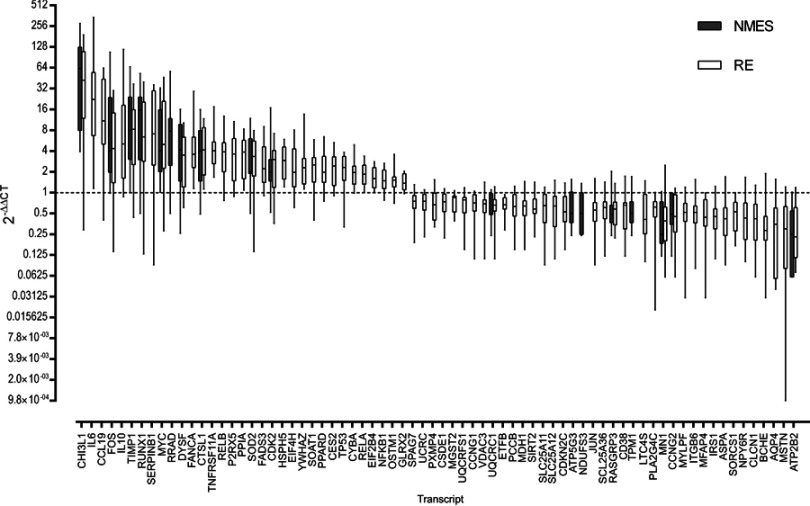
All genes significantly altered in expression following either transcutaneous neuromuscular electrical stimulation (NMES; 18 genes) or resistance exercise (RE; 68 genes) with a false discovery rate (FDR) <5%. Data are expressed as fold change from baseline, where the baseline value equals 1 (dashed line). Boxes denote median and interquartile range, whiskers are range. Magnitude of fold change (2^−DDC^_T_) are log values. Abbreviated gene names used in this figure are defined in Table S1.

**Figure 2:**
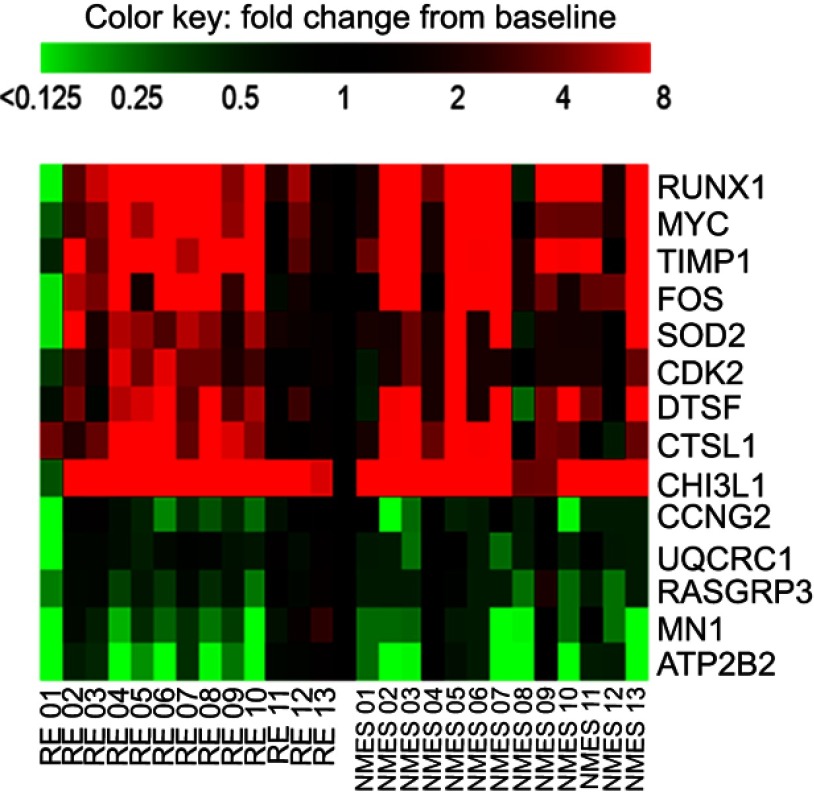
Heatmap demonstrating individual variation in response to transcutaneous neuromuscular electrical stimulation (NMES) and resistance exercise (RE) for the 14 transcripts that were significantly influenced by both interventions. Scale capped at 8-fold change, baseline value =1. Abbreviated gene names used in this figure are defined in Table S1.

Twenty-four hours after NMES, 18 mRNA transcripts were significantly changed in abundance, with 68 mRNA transcripts significantly changed in abundance 24 hours after RE (both FDR <5% compared to baseline, Figure 1). Changes in abundance of 14 gene transcripts were common to both NMES and RE,with no significant difference in the magnitude of fold change between groups for these common genes (*P*>0.05). The within-group variation in the response to NMES and RE is depicted in Figure 2. Mean fold change values for all targets measured are listed in Table S2 of the Supplementary materials.

The 14 transcripts significantly influenced by both NMES and RE perform a range of physiological roles, which are detailed in Table 2, accompanied by fold change data. Genes with physiological roles associated with muscle hypertrophy, growth, repair, regeneration, and anti-wasting, respectively (*RUNX1*, *MYC*, *TIMP1*, *FOS*, and *DYSF*), were upregulated following both NMES and RE. Other upregulated transcripts *CTSL1*, *CHI3L1*, *CDK2*, and *SOD2* have physiological roles relating to protein breakdown, anti-inflammatory action, cell cycle regulation, and antioxidant action, respectively. Downregulated transcripts *CCNG2*, *ATP2B2*, and *RASGRP3* are influential in cell cycle/signaling regulation. RASGRP3 also has a physiological role in cancer, as does *MN1*, with both of these transcripts downregulated after both interventions. Another transcript downregulated following both NMES and RE, *UQCRC1*, codes for a mitochondrial sub-unit.

**Table 2:** List of transcripts significantly altered in expression following a bout of NMES or RE

Target name	Full name	Physiological role	NMES		RE	
			Fold change	FDR %	Fold change	FDR %
(2^−ΔΔC^_T_)	(2^−ΔΔC^_T_)
Increased after both electrical stimulation and resistance exercise						
*CHI3L1*	chitinase 3-like 1	Protects muscle against inflammation^20^	84.4	<0.001	59.3	0.003
*RUNX1*	runt-related transcription factor 1	Upregulated by exercise. May prevent muscle wasting^21^,^22^	15.7	0.005	12.6	0.009
*MYC*	v-myc avian myelocytomatosis viral oncogene homolog	Muscle growth via ribosomal biogenesis^23^	10.3	0.011	11.5	0.006
*FOS*	FBJ murine osteosarcoma viral oncogene homolog	Transcription factor for pro-hypertrophy genes^24^,^25^	17.6	0.011	8.7	0.029
*TIMP1*	TIMP metallopeptidase inhibitor 1	Promotes cell proliferation, inhibits apoptosis. May promote angiogenesis^26^,^27^	16.7	0.024	11.2	0.007
*SOD2*	superoxide dismutase 2, mitochondrial	Antioxidant^28^	3.6	0.029	3.6	0.036
*DYSF*	dysferlin	Muscle repair/regeneration^29^	6.2	0.033	4.2	0.013
*CDK2*	cyclin-dependent kinase 2	Cell cycle regulation^30^	3.2	0.033	3.1	0.025
*CTSL*	cathepsin L	Protein breakdown^31^	5.2	0.027	5.3	0.003
Downregulated after both electrical stimulation and resistance exercise						
*MN1*	meningioma (disrupted in balanced translocation) 1	Cancer^32^	0.4	0.024	0.6	0.01
*CCNG2*	cyclin G2	Cell cycle^33^	0.6	0.024	0.6	0.016
*RASGRP3*	RAS guanyl releasing protein 3	Cell signaling^34^	0.6	0.024	0.6	0.036
*ATP2B2*	ATPase, Ca++ transporting, plasma membrane 2	ATP Pump controlling intracellular calcium level^35^	0.4	0.024	0.4	0.003
*UQCRC1*	ubiquinol-cytochrome c reductase core protein I	Mitochondrial sub-unit^36^	0.6	0.027	0.7	0.025

**Notes:** False discovery rate (FDR) <5% is the threshold for significance. Between group differences in fold change all *P*>0.05. 2^−ΔΔC^_T_, mean fold change from baseline corrected for expression of the internal control gene (HMBS).

**Abbreviations:** NMES, transcutaneous neuromuscular electrical stimulation; RE, resistance exercise.

## Discussion

This study describes altered expression of targeted mRNA transcripts 24 hours after a 30 minute bout of transcutaneous electrically evoked muscle contraction or voluntary muscle contraction in the quadriceps of matched cohorts of patients with COPD. The major findings are 1) RE influenced a substantially broader range of transcripts than NMES (68 vs 18, respectively); and 2) a smaller number of transcripts (representing 14 genes) responded similarly to the two interventions. Our conclusion is that the NMES intervention employed in this study was not sufficient to stimulate the same breadth of transcriptional response generated by maximal voluntary RE in COPD.

This is the first study to examine the influence of NMES on the expression of a broad range of mRNA transcripts and, furthermore, to compare this response to that following a bout of voluntary RE in well-matched groups of COPD patients. There is existing evidence of a transcriptional response to NMES in young non-weight trained individuals,^37^ but this had not previously been reported for patients with COPD. NMES has previously been shown to influence phosphorylation of p70S6K, a regulator of muscle protein synthesis,^38^ and influence muscle fiber size,^39^ suggesting that repeated bouts of NMES in COPD promote muscle fiber adaptation. More is known about the molecular changes in muscle following a bout of voluntary RE in COPD; expression of gene transcripts with functions relating to muscle protein synthesis and breakdown, myogenesis, transcription factors, and inflammation have previously been shown to respond to a bout of isokinetic resistance exercise in COPD patients^13^ in a manner comparable to that shown in our study.

### Breadth of transcriptional response to NMES and RE

NMES was performed at the highest tolerable intensity in order to maximize muscle fiber recruitment; however, the narrower range of gene transcription responses following NMES is likely to be a function of less muscle recruitment during NMES compared to RE. This is supported by the observation that the changes in mRNA abundance of the 14 common transcripts were similar between NMES and RE. A limitation of this study is that muscle tension development during NMES was not measured, but is known to typically be below 15% of maximal voluntary isometric force generation^10^ and might, therefore, be assumed to have been considerably lower than that produced during RE.^38^

The contraction provoked by NMES does not follow Henneman’s size principle^40^ of motor unit recruitment, rather NMES depolarizes larger motor neurones first, thus preferentially activating fast-muscle fibers (which tend to be on the periphery of the muscle bundle^41^), or may activate fibers in a spatially determined manner dependent on proximity to the skin-surface electrode.^42^ The genes differentially regulated by NMES may, therefore, have been influenced by the type of muscle fiber recruited during electrical stimulation; unlike RE, where all fiber types are likely to have been recruited.

NMES improves muscle strength and function across a range of stimulation frequencies,^9^,^11^,^43^–^47^ with efficacy dependent on stimulation intensity,^38^ which determines contraction force. Stimulation intensity is limited by the tolerance of the individual and, in healthy individuals, there have been reports of pain during stimulation.^48^ In health there have also been reports of NMES causing muscle damage and delayed onset soreness,^48^–^50^ comparable to that caused by eccentric RE,^10^,^51^ despite generating lower force.^52^ However, NMES protocols utilized in chronic disease rehabilitation, that generally elicit lower contraction forces, appear to be well tolerated^43^ and significantly increase muscle strength and mass, even when generating a force as low as 13% of maximal voluntary contraction.^38^

### Common responses

The transcriptional response of muscle to both NMES and RE was similar for 14 gene transcripts (Figure 2 and Table 2), whether these changes reflect mutual type II muscle fiber recruitment is unknown. Functions of shared upregulated transcripts were associated with muscle growth, repair and regeneration,anti-inflammatory/antioxidant action, and protein breakdown. In addition, there was common downregulation of gene transcripts associated with cell cycle, cancer, and mitochondrial function. Following both interventions, the most markedly upregulated mRNAs were *CHI3L1* and *RUNX1*. *CHI3L1* (chitinase-3-like protein 1) gene expression is known to be induced by contractile activity,^53^ and the protein is associated with myoblast proliferation^53^ and inhibition of the inflammatory response.^20^ *RUNX1* (runt-related transcription factor 1) may be protective against disuse atrophy,^21^ and there is a pronounced increase in expression when muscle is exercised after a period of immobilization.^54^ *RUNX1* may also be a target of *MYOD1*, which regulates myogenesis and skeletal muscle differentiation.^55^ Whilst the influence of any individual gene on muscle function or architecture is likely to be small, the strong induction of these two genes after both NMES and RE supports the notion that both interventions are influencing muscle cells towards a pro-growth state. We performed a pathway analysis on the 14 common genes using Ingenuity Pathway Analysis (IPA; QIAGEN, Redwood City, CA, USA www.qiagen.com/ingenuity). Due to the small number of transcripts, only a single cellular function (Cell Death and Survival) was identified by IPA as being significantly altered, with a relatively low level of significance (Figures S1 and S2).

The fully quantitative and highly sensitive RT-PCR technique employed in this study allows characterization of a wide range of expression values. Furthermore, the intervention groups were well matched for age, gender, and body composition, and adhered to a carefully planned study day protocol. There were more current smokers in the RE group. There is some evidence that cigarette smoke exposure may downregulate resting muscle protein synthesis rates in humans,^56^ and inhibit muscle signaling pathways in mice;^57^ however, in the current study there was no difference in fat-free mass between groups at baseline, and it was the RE group (who had the greater cigarette smoke exposure) who demonstrated the largest mRNA response to the interventions used in this study. Therefore, we are confident that the differences in gene expression observed after the two interventions were as a result of the contraction mode, rather than a characteristic of the two groups. We have considered the likely influence of the prior biopsy procedure on mRNA abundance 24 hours after muscle contraction. Evidence from healthy subjects in our own laboratory^14^ and other’s^17^ demonstrate no transcriptional changes in skeletal muscle after serial needle biopsy procedures in the absence of exercise, thus we are confident that the mRNA responses reported here are a reaction to muscle contraction, rather than the biopsy procedure per se.

## Conclusion

In conclusion, a novelty of this study is that it demonstrates a single bout of RE influences the expression of a far wider selection of genes than a single bout of NMES. However, there is a commonality of response for a small sub-set of gene transcripts. Based on our evidence, voluntary RE would appear to be the preferable mode of exercise intervention to elicit the largest muscle transcriptional response in stable COPD patients. However, NMES within the limits of comfort for patients does elicit a pro-growth transcriptional response. Further work is warranted to investigate the effect of repeated bouts of NMES and RE on molecular responses and physiological adaptation to a chronic intervention.

## Abbreviations list

ATP2B2, ATPase, Ca++ transporting, plasma membrane 2; BMI, body mass index; CCNG2, cyclin G2; CDK2, cyclin-dependent kinase 2; CHI3L1, chitinase 3-like 1; COPD, chronic obstructive pulmonary disease; CTSL1, cathepsin L; DEXA, dual energy x-ray absorptiometry; DYSF, dysferlin; FDR, false discovery rate; FFMI, fat free mass index; FOS, FBJ murine osteosarcoma viral oncogene homolog; HMBS, hydroxymethylbilane synthase; LDA, low density array; MN1, meningioma (disrupted in balanced translocation) 1; mRNA, messenger ribonucleic acid; MYC, v-myc avian myelocytomatosis viral oncogene homolog; NMES, transcutaneous neuromuscular electrical stimulation; p70S6k, ribosomal protein S6 kinase B1; PCR, polymerase chain reaction; RASGRP3, RAS guanyl releasing protein 3; RE, resistance exercise; RT-PCR, reverse transcription PCR; RUNX1, runt-related transcription factor 1; SD, standard deviation; SOD2, superoxide dismutase 2, mitochondrial; TIMP1, TIMP metallopeptidase inhibitor 1; UQCRC1, ubiquinol-cytochrome c reductase core protein I.

## Data Availability

The datasets used and/or analyzed during the current study are available from the corresponding author on reasonable request.
